# Tooth Bearing Tumors of the Jaw

**Published:** 1877-12

**Authors:** D. C. Hawxhurst


### TOOTH-BEARING TUMORS OF THE JAW.

BY D. C. HAWXHURST, M. D., D. D S.

I shall try to give you to-day, gentlemen, what is known up to the present date, on the subject of Dentigerous Cysts. These cystic tumors constitute a pathological anomaly of great interest although of rare occurrence.

There are many lesions that effect the mixillæ, which are found throughout the whole osseous system, and which effect the jaws, in common with the other bones of the body. Such tumors fall outside of the present discussion; leaving all such, I shall aim to make some intelligent observations on a special form of maxillary enlargement, that is due to the retention of a tooth in the bony cell where it was first developed, and its permanent inpaction there.

This species of tumors is not only confined in its position to the maxillæ. It arises exclusively in the *dental capsule* of an unerupted tooth and is to be regarded as among the rarer complications of dentition.

The impacted tooth not being able to erupt, forms the seat of a slowly increasing irritation which is followed by an accumulation of serum in the *capsule.* Such tumors sometimes attain to enormous size and assume a very formidable aspect. They have many points of interest to the practitioner, one of which is, their difficulty of diagnosis. This, conjoined with the fact that any one of us on any day is liable to be confronted by such a case, is a gaurantee of our deepest interest.

Dentigerous Cysts have some well marked characteristics whereby they may be distinguished from other enlargements of the maxillæ of different pathological origin.

Without attempting any extended description, I shall condense into the fewest number of words, such distinguishing traits as will in general enable us to discriminate between these tumors and other of the same locality.

I. Dentigerous Cysts generally occur in the region of an unerupted tooth of the regular dentition. The tooth appears to be absent from the arch, and there is no history of its extraction. If now, a tumour presents itself in this locality it should be looked upon with suspicion.

But sometimes though rarely the offending organ, is a supernumerary tooth, in which case there is absent one important means of diagnosis; since, if the regular number of teeth is present the practitioner will not have the same grounds for suspecting the inpaction of a tooth. Salter records two cases of Dentigerous Cysts dependent on the inpaction of supernumerary teeth.

I have also seen recorded two cases of cysts containing deciduous teeth, one by Salter and one by Heath. These are however rare exceptions. It is a rule that the incisted organ is a permanent tooth of the regular dentition.

II. These tumors are slow in their development. M. Forget describes a case that was fifteen years in progress, and another that had been of ten years duration. Another ran its course of development in six years. A case that fell under my own observation was operated upon at the end of three years, and might no doubt have gone three years longer before operation, without fatal results.

III. These tumors are generally painless in all their earlier stages, and are marked by mere fullness and heaviness during their later stages. As an exception to this rule, Forget records a case in which there was severe dental neuralgia, covering a period of ten years. In this case the tumor encroached upon the dental nerve. Ordinarily the individual is entirely unconscious of the existence of the tumor until its increase in size gives rise to deformity. And for a long time after that the case may go on with scarcely more than a slight sense of fullness.

IV. These tumors generally appear before the age of thirty. Yet in one case, recorded by Mr. Heath, a tumor developed at sixty. And in another recorded by M. Forget at fifty-six. They sometimes occur at an early age. In Mr. Ferns' case, recorded in Mr. Heath's work on Injuries and Diseases of the Jaws, the tumor was developed at the age of thirteen. He records another case which developed in a child of four years of age.

V. These tumors sometimes exhibit a crackling on pressure. This is present only when there is attenuation of the osseous wall that covers the impacted tooth, and could therefore appear only after the tumor had reached an advanced stage of evolution.

VI. These tumors contain, along with the tooth, a quantity of serous fluid, which may or may not degenerate into pus according as there is more or less inflammation. Ordinarily there are to be seen floating in it, crystals of cholesterine, which sparkle with a yellow light as they hang suspended in the yellow, glossy serum. Without further remarks, I shall describe to you a case that occurred in my own practice in 1875.

A boy of twelve years of age was sent to me with great tumefaction of the right cheek. On opening the jaws the right side of the mouth was seen to be occupied by a bulging tumor, the base of which extended two and one-half inches from side to side, reaching from the cuspid tooth in front to the maxillary tuberosity, which it included. Extending beyond the medium line it obliterated the concavity of the hard palate except a deep groove between it and the left upper teeth. The facial and zygomatic surfaces of the jaw, which are naturally hollowed, bulged outward so as to produce much external deformity. Three permanent teeth were absent from their place in the jaw which should have been erupted several years previously. A part of the tumor projected downward by the side of the tongue between it and the lower teeth. At a point where it was indented by the lower teeth it gave out a slight but apparently superficial discharge of pus.

This tumefaction had been in progress three years from the time of its first discovery. It had been lanced by Dr. O. H. Coller a few months after its first manifestation; had discharged slightly during a period of nearly a year, and had been closed. It began as a simple enlargement on the hard palate opposite the still vacant place of the first permanent molar. It grew slowly and steadily without pain or inflammation, or other inconvenience than its size, in which respect, was like most tumors of its class; though some tooth bearing tumors have been both painful and rapid in their formation.

The parents believed that their boy was afflicted with a cancer, as several doctors had so named the tumor.

I should say that Dr. Coller had rightly opposed this view, though there was much in the external appearance of the swelling to support the view that it was a case of malignant disease. But the youth and excellent health of the patient, along with the absence of cancer in the family history, and of infiltration of the surrounding tissues contra-indicated malignant disease. While on the other hand there was evidence pointing unmistakably to Dentigerous Cysts. The absence of pain in the tumor, the length of time during which it had been in progress and especially the absence of three permanent teeth at its seat, were all circumstances calculated to point attention in this direction. In this Dr. Coller concurred with me.

Having made all things ready for operation, I inserted a groove needle and had the satisfaction of seeing a fluid resembling serum, issue as soon as the needle had penetrated a thin osseous shell and made its way to the interior. This attenuated osseous covering was additional evidence that the diagnosis was now complete.

I had determined to make the operation entirely inside the mouth. The patient was under the influence of cholroform and assistants were at hand.

A large section of the tumor was removed by two clips of a pair of bone forceps dissecting off the mucus membrane and pressing it back in flaps, I cut away the osseous covering of the cysts to the extent of one and one-half inches one way and two the other with a pair of gouge forceps. I carved away not only all remains of the cyst, but some of the tumefied bone external to it, with a view of saving deformity and hastening a return of the features to their normal state.

During the operation a large but imperfectly formed molar tooth was taken from the cyst. Its grinding surface was free and formed a part of the wall of the fluid-containing sac. The boy made a rapid recovery under the care of Dr. Pease of Waukeskma Center.

Twenty-three months after the operation I saw this patient and found the parts in a state of perfect health. The external deformity had almost entirely disappeared.

It must now be recalled that on my first examination three teeth of the permanent dentition,—the first molar, the second bicuspid and the canine, were absent and it will be remembered that but one tooth was taken away from the tumor during the operation. On making this examination the missing second bicuspid was found to have erupted in an oblique or nearly horizontal position from the cicatrix of the cyst.

The cuspid which was also primarily absent, now lay under the gum ready to erupt immediately beneath the lip at a distance of two teeth and a half forward of its normal position. The eruption of these two teeth was a matter of some importance since they, along with the first permanent molar taken from the cyst itself make up the compliment of the complete permanent dentition.

I found the boy now fourteen years of age, perfectly healthy and robust with no signs of a return of the tumefaction.

TREATMENT: The treatment for these tumors consists in the removal of the contents of the cyst, which is a tooth or some abnormal development of dental tissue, surrounded by a serous fluid. The cyst itself need not be dissected out, it will soon shrink and disappear on the removal of the tooth, which caused its development. In a few rare cases, it has been injected with astringent and stimulating fluids to arrest the serous discharge.

The expanded bony wall of the cyst should be opened up sufficiently to permit healing by granulation from the bottom. The excessive bony formation is, in almost every case, rapidly absorbed. It may however be cut away, if it be very abundant, as was done by Nelaton, or crushed in, as was done by Tomes.

In the case which I have related I should have removed less of tissue and trusted more to subsequent absorption had I been better acquainted with the experience of Tomes and other European surgeons. There is this disadvantage however attendant up the opposition of crushing in the walls of the cyst. The deformity is much less rapidly relieved.

The operation may generally be made entirely within the mouth, and the more especially if it be performed before much enlargement has taken place. An early diagnosis is therefore highly desirable.

So far as I know, only one case has been recorded in which the above treatment did not procure complete and permanent relief. A record of such invariable success following any mode of treatment is a gaurantee of its correctness. But Salter describes one case in which the wound became the seat of a fibrous tumor after the removal of the cyst. This was dissected away and did not return.

As you will see, I have made no attempt to describe those other cysts and tumors of the maxillæ that do not originate in impacted teeth; nor have I included several kinds of tumors that find their point of departure in morbid processes occurring in tooth pulps at an early stage of their development.*

